# The genome of the relict earless monitor lizard, *Lanthanotus borneensis*, and the Toxicofera hypothesis

**DOI:** 10.1186/s12915-026-02552-4

**Published:** 2026-03-05

**Authors:** Magnus Wolf, Axel Janke, Krister T. Smith

**Affiliations:** 1 Senckenberg – Leibniz Institution for Biodiversity and Earth System Research, Senckenberg Biodiversity and Climate Research Centre, Georg-Voigt-Strasse 14-16, Frankfurt Am Main, Germany; 2 Institute for Evolution and Biodiversity (IEB), University of Muenster, Huefferstrasse 1, Muenster, Germany; 3 Goethe University Frankfurt, Faculty of Biosciences, Institute for Ecology, Evolution and Diversity, Max-von-Laue-Strasse 9, Frankfurt Am Main, Germany; 4 Senckenberg – Leibniz Institution for Biodiversity and Earth System Research, Senckenberg Research Institute and Natural History Museum Frankfurt/M., Senckenberganlage 25, Frankfurt Am Main, 60325 Germany

**Keywords:** Squamata, Toxicofera, Venom, Genome, Incomplete lineage sorting, Deep divergences, Positive selection

## Abstract

**Background:**

The earless monitor lizard, *Lanthanotus borneensis*, is a unique living fossil restricted to the island of Borneo and a possible key to understanding the evolution of the venom delivery system and secondary adaptation to water in lizards and snakes (Squamata).

**Results:**

We sequenced and de novo assembled the genome of *L. borneensis* to a total size of 1.5 Gbp, 975 contigs with an N50 of 52 Mbp and an L50 of 9. The genome completeness is estimated to be 93% based on the Sauropsida OrthoDB core gene set. A genome-wide set of Lepidosauria orthologs was compiled to reconstruct and date their phylogeny, resulting in 966 protein-coding sequences amounting to a concatenated alignment of 356 kbp with 188 kbp parsimony-informative sites. Based on this phylogenomic analysis, one of the largest of its kind yet conducted for Squamata, we identified that a Toxicofera clade (comprising Serpentes, Anguimorpha, and Iguania) is supported by a plurality of gene trees, but critically, support for relationships within Toxicofera is almost equally distributed amongst the three possible topologies. Our tree-dating confirms a rapid divergence of all major squamate clades within the first 10% of squamate history, which may have contributed to rampant incomplete lineage sorting. While we did not identify positive selection on genes associated with venom components at the base of Toxicofera, our analyses found strong positive selection on the giant protein titin throughout the main clades of Toxicofera and especially in snakes. Genome-wide heterozygosity is low (H_O_ = 0.0004), as is the effective population size towards the present.

**Conclusions:**

Future studies of the evolution of the venom delivery system in Toxicofera require a “true” species tree but also individual gene trees due to incomplete lineage sorting and the concomitant potential for hemiplasy. Titin—a key component of striated muscle elasticity—emerges as a target for future evolutionary studies in Toxicofera and especially in wide-gaped snakes (“Macrostomata”). The low observed genome-wide heterozygosity and the low but stable effective population size of *L. borneensis* during the large-scale habitat fluctuations on Sundaland in the Quaternary suggest an unexpected resilience to environmental perturbations but also a potentially lowered adaptive potential of this isolated lineage.

**Supplementary Information:**

The online version contains supplementary material available at 10.1186/s12915-026-02552-4.

## Background

The earless monitor lizard, or Borneo Earless Monitor, *Lanthanotus borneensis*, is a highly elusive, semi-aquatic reptile endemic to northwestern Borneo and the sole extant representative of the family Lanthanotidae. Long regarded as one of the least-understood lizards due to its secretive behavior and limited distribution [1–3], this species occupies a unique evolutionary position as the sister taxon to the family Varanidae (monitor lizards). As a phylogenetic relict, the species offers a rare window into the evolutionary history of anguimorph lizards, with implications for understanding deep-time divergence events within Squamata.

The isolated taxonomic position of *Lanthanotus borneensis* makes it a critical comparative outgroup for studying the genomic innovations in Varanidae, known for their rapid diversification and complex physiology [4–7]. It may offer unique insights not only into squamate phylogeny but also into secondary adaptation to aquatic environments and the genomic architecture underlying ancient lineages. Furthermore, the species’ probable low metabolic rate, presumed environmental sensitivity, and specialized ecology [3, 8, 9] present an opportunity to investigate conserved and lineage-specific genomic features associated with environmental resilience, niche specialization, and physiological stasis. Sequencing and analyzing the *L. borneensis* genome may thus illuminate both the tempo and mode of genomic evolution in ancient squamate lineages.

Moreover, *Lanthanotus borneensis* is considered endangered and in urgent need of further study. In fact, it is so poorly known that an initial proposal to place it on CITES Appendix I [10] was rejected for want of data supporting the threat status, and it was subsequently, on January 2, 2017, placed on CITES Appendix II on the criterion of limited geographic range [11]. Its geographic range, and probably population size, is decreasing [11]. Intensive surveys in localities in which it had previously been encountered have not produced recent sightings. Certainly, suitable habitat is increasingly restricted by deforestation [11]. Hence, inferences concerning population history may help inform conservation decisions in this phylogenetically isolated species.

Knowledge of the genome of *Lanthanotus borneensis* is limited. Wiens et al. [12] and Reeder et al. [13] reported data on 42 (of 46 targeted) nuclear genes of this species. Streicher and Wiens [14] sequenced 3073 nonexonic ultraconserved elements (UCEs); based on the same sequencing effort, Streicher and Wiens [15] gave 3256 UCEs, comprising 797,632 bp.

As an anguimorph lizard, *Lanthanotus borneensis* is part of Toxicofera [16], the clade comprising Iguania, Anguimorpha, and Serpentes and hence including all known venomous reptiles and their closest non-venomous relatives. Toxicofera achieved prominence with the seminal paper by Fry et al. [17], and although its composition was heterodox when first proposed, its historical reality is no longer considered contentious (e.g., [18]). Yet, there remain open questions concerning the interrelationships of Iguania, Anguimorpha, and Serpentes. There is a tendency to infer with increasing support that Iguania and Anguimorpha are sister taxa and together form the sister taxon of Serpentes [12, 13, 15, 19–21], and Singhal et al. [21] considered the matter of their interrelationships to be “resolved.” Yet only a limited portion of the squamate genome has been studied, and more modest support for the “resolved” topology has been discovered using genome-scale data [6]. Understanding the relative position of these taxa is important for broader inferences concerning the evolution of the venom delivery system—including glands and peptides—in Toxicofera. Hence, the genome will contribute not only to conservation and comparative genomics but aid in resolving key questions in the evolution of reptilian venom systems.

To clarify reptilian relationships and to provide a data source for future phylogenomic and toxin studies, we sequenced using PacBio to 78 × coverage and de novo assembled the first genome of the Borneo earless monitor, *Lanthanotus borneensis*. It also allows us to get a glimpse into the genomic diversity and past demography of this species, providing an important contribution to the conservation of this endangered and otherwise poorly known species.

## Results

### Genome assembly

**Fig. 1:**
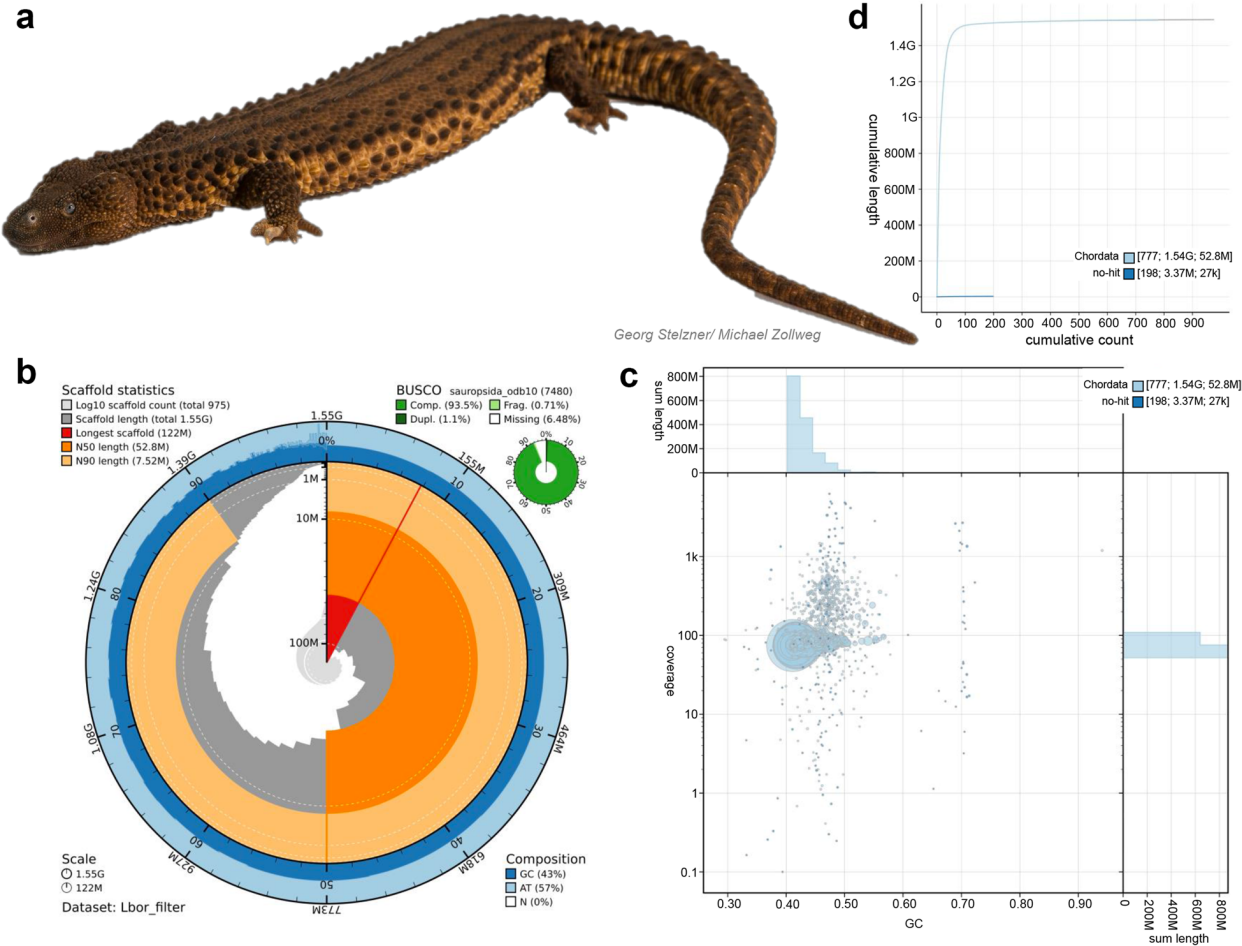
Assembly characteristic and quality analyses for the de novo genome of *Lanthanotus borneensis*. **a** Male individual of *Lanthanotus borneensis* whose genome is studied in this paper. **b** Snail plot summarizing genome assembly metrics, including scaffold length distribution, N50, and GC content. The outer ring displays the cumulative scaffold lengths sorted from largest to smallest. BUSCO completeness scores are shown in the outer green circle, based on the sauropsida_odb10 dataset. **c** Blob plot depicting the individual scaffolds as circles with size indicating their total length and color depicting taxonomic hits in the NCBI nr database. Positions on the x- and y-axis indicate coverage and GC content, respectively. Bar plots depict the total sum of the depicted scaffolds at the specific positions on the main graph. **d** Cumulative plot showing the sum of scaffolds assigned to respective NCBI nr hits, belonging either to the phylum Chordata or “no-hit” in this case. Photo of *L. borneensis* created and provided by Georg Stelzner and Michael Zollweg. Plots were generated using BlobToolKit [24]

PacBio CLR sequencing resulted in 11,886,802 circular consensus reads from which a de novo genome was assembled (see Fig. 1, Table 1) [22]. Illumina short-read sequencing resulted in 713,991,672 reads. The resulting assembly is 1,545,120,326 bp in size and contains 975 contigs of which 9 amount to the L50 value (Fig. 1b). Contig N50 reached 52,824,789 bp and the assembly contained no gaps and a GC content of 42.9%. The BUSCO core gene set identification based on the Sauropsida OrthoDB_10 collection [23] resulted in 93% complete, 0.7% fragmented, 1.1% duplicated, and 6.5% missing core genes, indicating a high completeness of the assembly. Read back-mapping rate to this assembly was 97.6% with a mean coverage of 78.4 × and 97.9% and 67 × for the long and short reads, respectively (Fig. 1c). After contamination filtering, all scaffolds could be assigned to the phylum Chordata, although a number of small scaffolds, 3.4 Mbp in sum, returned no hits in the NCBI Nucleotide database (Fig. 1d).

**Table 1:** Genome data statistics sequenced during this work

**Raw read statistics**	
No. long reads	11,886,802
Mapped long reads (%)	97.56
Mean long read coverage (x)	78.37
No. short reads	713,991,672
Mapped short reads (%)	97.87
Mean short reads coverage (x)	67
**Assembly statistics**	
No. contigs	975
No. contigs (> 50 kbp)	284
Contig L50	9
Contig N50 (bp)	52,824,789
Total length (bp)	1,545,120,326
GC (%)	42.97
No. of N’s per 100 kb	0
Total interspersed repeats (bp)	632,221,132 (40.91%)
Genome-wide heterozygosity	0.00042
**BUSCO completeness**	
Clade: Sauropsida	C: 93.5%, D: 1.1%
	F: 0.7%, M: 6.5%
	n: 7480

*BUSCO *Benchmarking Universal Single Copy Orthologs, *C *complete, *S *single copy, *D *duplicated, *F *fragmented, *M *missing. See also Additional file 1: Fig. S1.

### Phylogeny

**Fig. 2:**
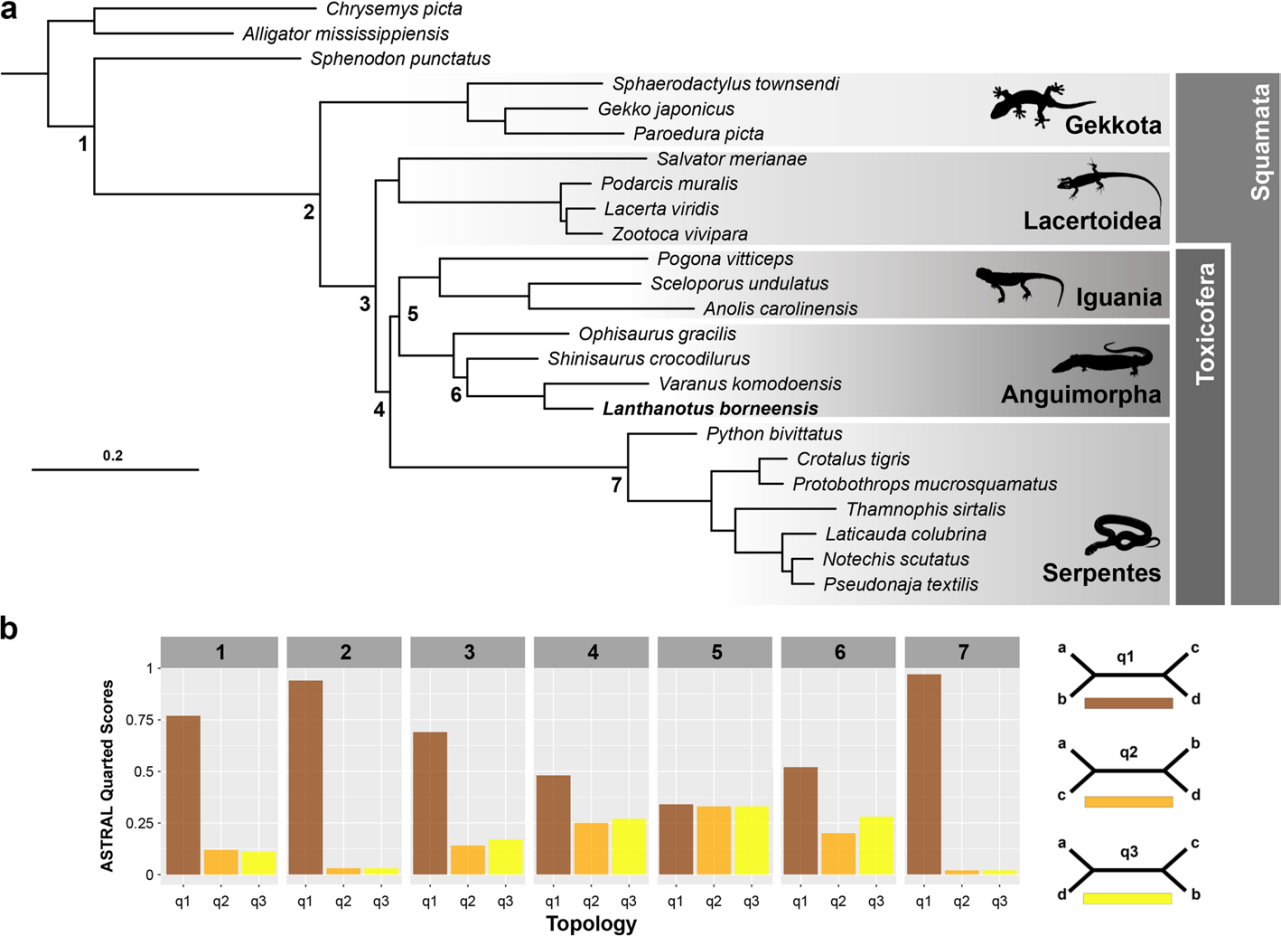
Maximum likelihood phylogenomic tree reconstruction of Squamata. **a** Species tree constructed by IQTree based on a concatenated alignment of 966 single-copy orthologs amounting to a total of 356 kbp and 188 kbp of informative sites. UFBootstrap values were 100% for every branch. Gekkota is the earliest divergence in Squamata (node 2), followed by Lacertoidea (node 3). The clade Toxicofera was recovered at node 4, in which Serpentes is the sister taxon to a clade formed by Iguania and Anguimorpha (= Dracomorpha). Within Anguimorpha, *Ophisaurus gracilis* (Neoanguimorpha) is the sister taxon to a monophyletic Paleoanguimorpha, comprising *Shinisaurus crocodilurus* and *Varanus* + *Lanthanotus*. **b** Quartet scores of selected branches across the MSC tree. Branches 1–7 were analyzed for the number of gene trees supporting one of the three possible unrooted topologies (q1–q3). Node 5 received nearly equal quartet scores for all three alternative topologies, i.e., for Iguania + Anguimorpha (preferred in slightly more gene trees), Iguania + Serpentes, and Anguimorpha + Serpentes

A maximum likelihood phylogenetic tree was constructed based on 23 publicly available genome sequences and the genome constructed in this study (Fig. 2a). After ortholog calling and extensive filtering, 966 shared single-copy orthologous amino acid sequences were used to concatenate a multiple sequence alignment of 356 kbp with 188 kbp parsimony-informative sites. Further phylogenetic work was conducted using the large, concatenated alignment of amino acid sequences.

Model comparison incorporated in IQTree returned the JTT + F + R6 model as the best fit. Conducting 1000 ultrafast bootstrap replications resulted in all branches being supported by 100%. The first species to diverge was *Sphenodon punctatus* (Tuatara). Afterwards, the geckos (Gekkota) split, followed by Lacertoidea, including the family of true lizards (Lacertidae) and the Tegu (*Salvator merianae*, Teiidae) in our sampling. Toxicofera, comprising the Serpentes, Anguimorpha, and Iguania, forms a monophyletic group with the snakes forming a sister clade to the latter groups [Iguania + Anguimorpha = Dracomorpha sensu [68]]. The earless monitor lizard (*Lanthanotus borneensis*) is deeply nested as the closest relative to the Komodo Dragon (*Varanus komodoensis*) within the Anguimorpha.

To further explore genetic conflicts, maximum likelihood gene trees were constructed independently and their resulting topologies were compared to the main species tree as quartet scores generated by ASTRAL-III [25] (Fig. 2b). Quartet scores, as their name implies, are concerned with the relative support amongst loci for the interrelationships of four lineages. These are, for any particular node (bifurcation), the two lineages defining that node together with the two subjacent (rootward) lineages. For any four lineages, there are three unrooted networks connecting them, which, after the quartet with the greatest relative support (quartet 1, q1), are arbitrarily labeled quartet 2 (q2) and quartet 3 (q3). The value associated with each quartet is the proportion of examined loci that support that particular unrooted network.

Seven main branches relevant to the Toxicofera clade and the earless monitor lizard were explored for their frequency of alternative topologies. The nodes closely above, at, or below Toxicofera (3–5) showed the highest number of alternative topologies in the collection of gene trees. The quartet topology analyzed at node 3 ((Toxicofera, Lacertoidea), (Gekkonidae, *Outgroup*)) appeared in q1 = 69% of the cases while alternative topologies were found in q2 = 14% and q3 = 17% of the gene trees. The quartet scores addressing branch 4 ((Dracomorpha, Serpentes), (Lacertidae, *Outgroup*)) suggest that q1 = 48% of the gene trees agree with the species tree while q2 = 25% and q3 = 27% display alternative topologies. Branch 5 located at the split of the Anguimorpha and Iguania ((Anguimorpha, Iguania), (Serpentes, *Outgroup*)) indicated an almost even distribution of quartet topologies with q1 = 34% supporting the species tree while q2 = 33% and q3 = 33% support alternative topologies. It is therefore branch 5 that directly concerns the interrelationships of the three toxicoferan clades, and it shows that a nearly identical number of genes support the alternative sister-group relationships (Anguimorpha, Iguania), (Anguimorpha, Serpentes), and (Iguania, Serpentes), with a slight preference for the first topology. In sum, a plurality of genes (48%) support the Toxicofera clade, but relationships within Toxicofera are less certain.

Node 6 comprises Old World anguimorphs, a clade called Paleoanguimorpha [26]. While the topology in Fig. 2a is well supported by a majority of genes, alternative topologies in which the neoanguimorph *Ophisaurus gracilis*, or a clade comprising *O. gracilis* and *Shinisaurus crocodilurus*, is sister to *Varanus* and *Lanthanotus* each receive support from about one-quarter of the genes.

### Divergence times

**Fig. 3:**
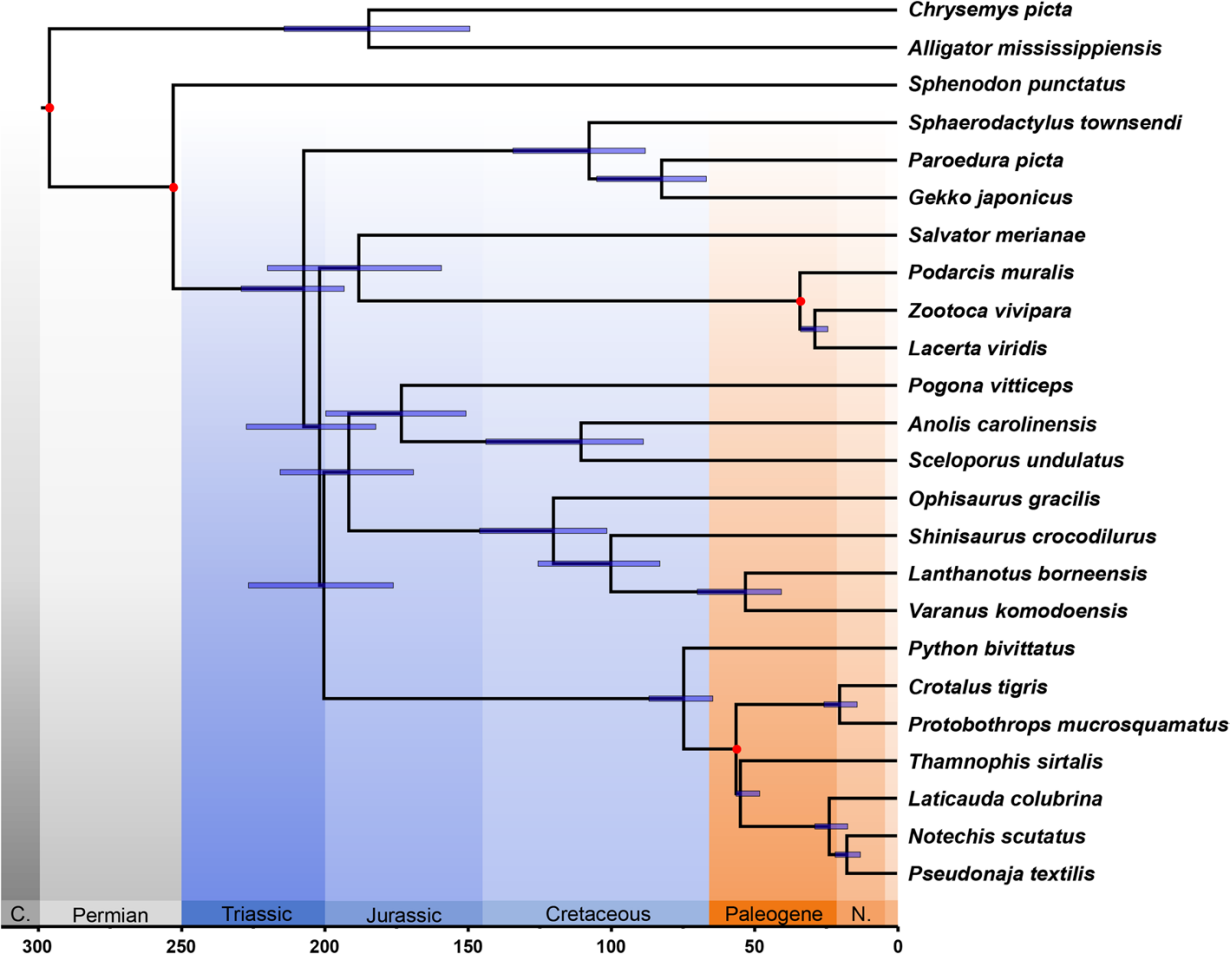
Divergence time estimates for Squamata. Estimates use four calibration points and are based on the LSD2 method from IQTree. Tree topology is based on Fig. 1, and an amino acid alignment of 966 single-copy orthologous sequences totaling to 356 kbp of sequence information. Calibration points can be found in Table 3. Major squamate clades originated in a short period between the Late Triassic and the Early Jurassic, which may explain the extent of incomplete lineage sorting and perhaps introgression in Toxicofera. The earless monitor lizard (*Lanthanotus*) diverged from the true monitor lizards (*Varanus*) by the latest Cretaceous

A calibrated tree was generated using the LSD2 method and was based on the previously generated maximum likelihood species tree as well as four fossil calibration points (Fig. 3, Table 1). Based on the oldest calibration point restricting the early Diapsida split, *Protorosaurus speneri* [27, 28], the tree ranges from the beginning of the Permian 295.9 Ma to the present. The basal split in Lepidosauria, separating the living fossil *Sphenodon punctatus* from Squamata (node 1 in Fig. 2), was also predefined to be 252.7 Ma [27, 28].

The basal divergence in Squamata, separating geckos from the remaining squamates (node 2 in Fig. 2), occurred in the Late Triassic, 207 Ma. This divergence was followed in short order by a series of further splits that define the major squamate clades (Lacertoidea, Serpentes, Iguania, Anguimorpha): the split between Lacertoidea and Toxicofera (node 3) at 202 Ma, the basal split between Serpentes and Iguania + Anguimorpha (node 4) at 200 Ma, and slightly later the split between Iguania and Anguimorpha (node 5) at 191 Ma. Younger divergences within these main clades include the split between Teiidae (represented by *Salvator merianae*) and Lacertidae at 188 Ma, the split between pleurodont and acrodont Iguania at 173 Ma, the split between *Python* and higher snakes (Caenophidia) at 75 Ma, and the split between Paleoanguimorpha and Neoanguimorpha at 120 Ma. *Varanus* and *Lanthanotus* split at 53 Ma in the early Eocene, with a 95% confidence interval of 70–40 Ma.

The overall character of these divergences—the origin of Squamata in the Late Triassic and rapid divergence of major clades in the Early Jurassic—is not substantially different when the divergence time estimates are based on a larger number (nine) of calibrations (Additional file 1: Fig. S2, Table S2). *Varanus* and *Lanthanotus* split at 52 Ma in the early Eocene, a value virtually indistinguishable from the value based on fewer calibrations, and the confidence interval does not extend down into the Cretaceous at all.

### Demographic history

**Fig. 4:**
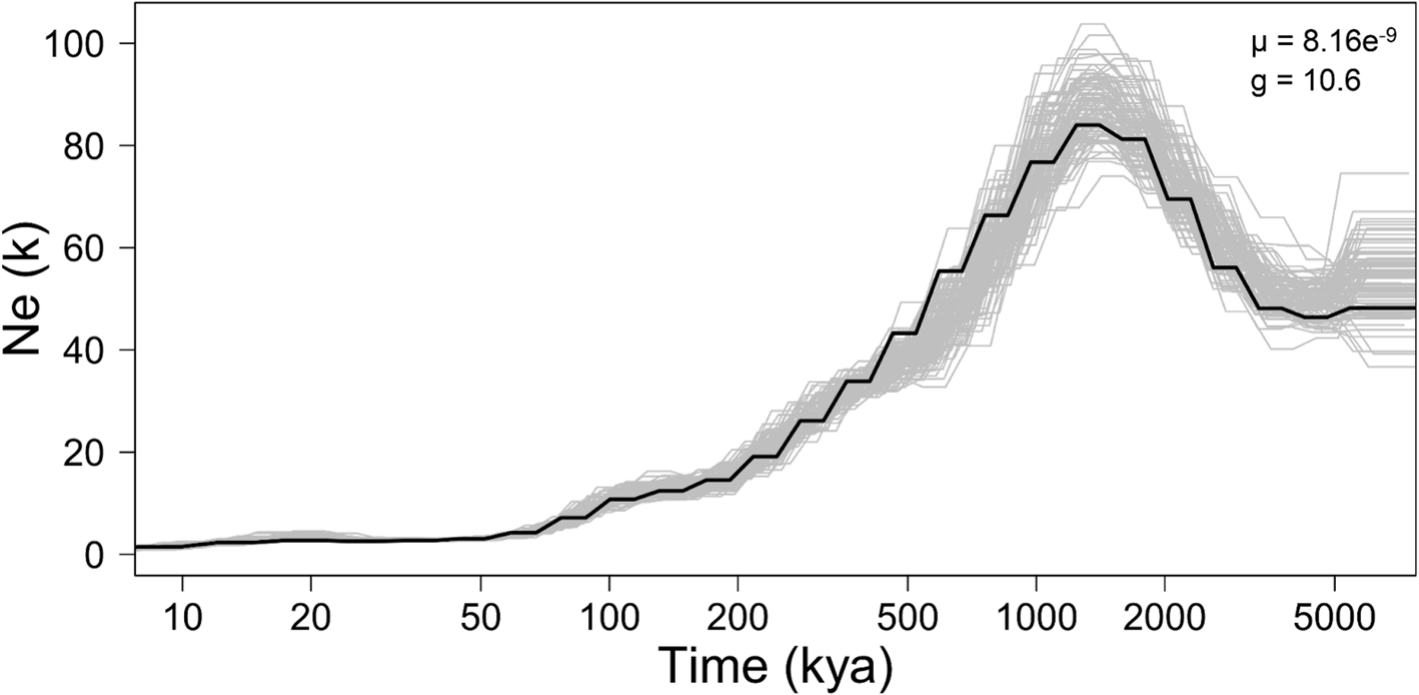
Bootstrapped PSMC analysis of *Lanthanotus borneensis*. The analysis assumes a mutation rate m of 8.16 × 10^−9^ and a generation time of 10.6 years. Gray lines show the bootstrap replications; the black line, the original population history based on the full dataset in kNe and kya. After a maximum at about 1.5 Mya, Ne drops dramatically but remains stable since about 75 kya

A PSMC (pairwise sequentially Markovian coalescent) analysis from this wild-caught individual (Fig. 4) modeled a total demographic history from 5 Ma to 10 kya. After an initial increase from 5 to 1.5 Ma and reaching an effective population size (Ne) maximum at ~ 80,000 individuals, population size gradually declined until reaching a stable value at 75 kya; bootstrap pseudoreplicates (gray lines) deviate very little from the overall result, especially in the latter interval. During this stable phase, *Lanthanotus borneensis* shows an Ne of about 5000 effectively breeding individuals. Ne sizes are typically dependent on a variety of factors and tend to be ten times larger in nature [29]. Using a smaller number of bins necessarily decreased the temporal resolution but did not change the overall pattern of population history (Additional file 1: Figs. S3, S4).

Observed genome-wide heterozygosity, measured as heterozygous sites per total number sites (H_O_), was 0.0004 (= 0.04%).

### Positive selection and functional enrichment

In studying evidence for selection, *ω* is defined as *d*_*N*_/*d*_*S*_, or the ratio of non-synonymous to synonymous substitution rates. If there are many more non-synonymous substitutions with respect to synonymous ones, there is evidence for positive or diversifying selection, because non-synonymous substitutions produce phenotypic change whereas synonymous substitutions do not. In contrast, if there are many more synonymous substitutions with respect to non-synonymous ones, there is evidence for negative or purifying selection that weeded out phenotypic variants.

**Fig. 5:**
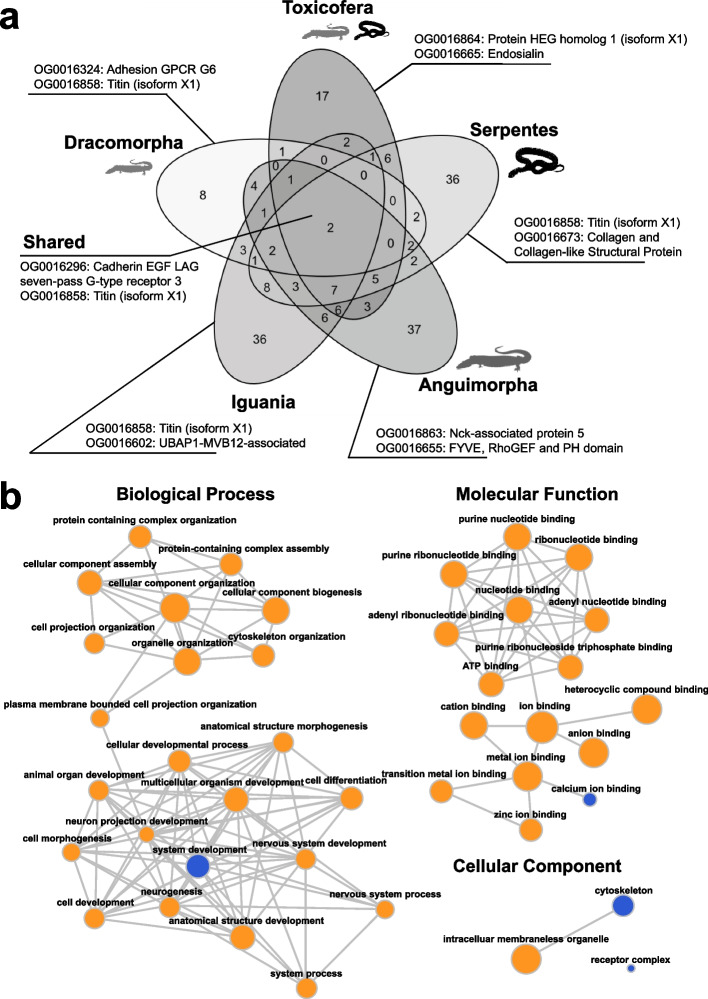
Positive selection and functional enrichment of genes in Toxicofera. **a** Venn diagram showing degree to which positively selected genes are unique or shared by more than one branch. Although the taxonomic composition of the clades is overlapping, the branches that subtend them are independent. Two orthogroups are identified as being positively selected in all five branches, including titin (isoform X1). **b** Interactive REVIGO graph describing functional term enrichment amongst positively selected genes. These are grouped here into those associated with biological processes, molecular function, and cellular components. All dots are positively selected functions; the blue ones are enriched functions compared to the overall set of orthologs we used in the phylogenetic analysis. Circle size indicates how often these functions appear in this overall set

A large number of positively selected genes was identified (Additional files 3–7). Many genes have very high values of *ω* (> 1000), but not necessarily high EBF or LRT values. The top two (ranked by LRT) for each branch are shown in Table 2. With respect to the five main branches comprising Toxicofera (Toxicofera, Dracomorpha, Iguania, Anguimorpha, Serpentes), we find that most are unique to one of the three mutually exclusive clades (Iguania, Anguimorpha, or Serpentes), with the number of genes nearly evenly distributed between them (Fig. 5a). We did not identify orthogroups closely associated with known venom components in Toxicofera [e.g., 30]. While orthogroups OG0016665 (LRT 23.07, EBF 7) and OG0016316 (LRT 5.42, EBF 15) include C-type lectin-like proteins, it appears that the identified orthologs belong to different families than the common components of advanced snake venoms [31, 32].

**Table 2:** Top two genes showing positive selection in main branches of Toxicofera and shared by all

Clade name	OG	Annotation	*ω*	EBF (> 5)	LRT
Shared	OG0016296	Cadherin EGF LAG seven-pass G-type receptor 3	2.42–275.75	4–61	6.30–17.82
	OG0016858	Titin (isoform X1)	3.21– > 1000	65–1564	9.03–104.55
Toxicofera	OG0016864	Protein HEG homolog 1 (isoform X1)	297.84	33	126.35
	OG0016665	Endosialin	> 1000	7	23.07
Dracomorpha	OG0016324	Adhesion G-protein coupled receptor G6	> 1000	166	643.80
	OG0016858	Titin (isoform X1)	31.18	86	22.64
Iguania	OG0016858	Titin (isoform X1)	25.52	65	104.55
	OG0016602	UBAP1-MVB12-associated	> 1000	10	51.29
Anguimorpha	OG0016863	Nck-associated protein 5	575.23	16	56.87
	OG0016655	FYVE, RhoGEF, and PH domain	172.02	7	35.16
Serpentes	OG0016858	Titin (isoform X1)	> 1000	1564	57.33
	OG0016673	Collagen and collagen-like structural protein	34.39	17	55.42

*OG *orthogroup label, *ω *dN/dS ratio, *EBF *empirical Bayes factor, *LRT *likelihood ratio test value.

Unexpectedly, we also discovered two orthogroups that underwent positive selection on every one of the five main branches of Toxicofera, including titin (isoform X1) (Fig. 2a; Table 2). A major component of striated muscle, titin makes a critical contribution to muscle elasticity and has the largest molecular weight of any known protein [33]. Overall, there was significant positive selection on genes related to tissue and organ differentiation as well as cellular structure (Fig. 5b).

## Discussion

### The genome of *Lanthanotus borneensis*

The long-read sequencing data allowed to assemble the genome to a high contiguity (N50 = 53 Mbp) which is comparable to or even surpasses many of the published genomes for other Anguimorpha, e.g., the Komodo Dragon *Varanus komodoensis* (N50 = 24 Mbp [6]; N50 = 23 Mbp [34]), the Asian Glass lizard *Ophisaurus gracilis* (=*Dopasia gracilis*; N50 = 1.27 Mbp [35]), and the Guatemalan Beaded Lizard *Heloderma charlesbogerti* (N50 = 1.34 Mbp [36]), although recently, a number of genomes have been constructed to chromosome-level using additional Hi-C technology like the Chinese Crocodile Lizard *Shinisaurus crocodilurus* [37], the San Diegan Legless Lizard *Anniella stebbinsi* (VGP 2025), and the Southern Alligator Lizard *Elgaria multicarinata* (VGP 2023).

BUSCO completeness values also fall within the expected range from other Anguimorpha and reptiles in general as compared in Chetruengchai et al. [38] and Dyson et al. [36]. We further found no signs of contamination in the constructed genome, and the GC content (43%) is on the lower end of other genomes of Anguimorpha [36]. Repeat annotation further revealed a total level of repeat coverage (41%) that is above other genomes of the genus *Varanus* [6, 38], but below other Anguimorpha genomes [35–37]. Given these genome characteristics in comparison to other published genomes, the new assembly qualifies as a high-quality reference genome that will aid in future genomic studies of the clade.

### Squamate phylogeny and the Toxicofera triumvirate

Since Camp [39], Iguania was considered to be the first branch of the squamate tree (e.g., [40–43]). This conclusion was challenged by Vidal and Hedges [16] and Fry et al. [17], who determined that Iguania is more deeply nested within Squamata, forming a clade together with Anguimorpha and Serpentes, a topology that every subsequent phylogenetic analysis of molecular data has supported. Vidal and Hedges [16] introduced the new clade name Toxicofera for the triumvirate Iguania + Anguimorpha + Serpentes. Most phylogenetic and phylogenomic (e.g., [17, 20, 21, 44, 45]) as well as phylotranscriptomic [19] work has found Iguania and Anguimorpha to form a clade within Toxicofera. That is to say, according to these studies, Serpentes is the first branch of Toxicofera. Mongiardino Koch and Gauthier [46] compared likelihood support for the Toxicofera topology with the traditional topology for each of 46 genes [13] and also found that the phylogenetic signal in most of these genes was low; they furthermore identified compositional bias with the genes of Serpentes and Iguania being somewhat richer in AT (by 1.3%) than other squamates and proposed that increased evolutionary rates in these two clades could be responsible for the recovery of Toxicofera. Singhal et al. [21], in particular, took each of several thousand loci of nonexpressed UCEs and examined the difference in log-likelihood between the maximum likelihood topology and the topology with the next-highest likelihood. They found that 88% of loci support this topology, amongst loci for which the difference in log-likelihood between the most-likely and next-most-likely topology was > 2, conventionally a threshold for strong support for the most-likely topology. Accordingly, they considered the matter to be resolved. Zaher et al. [47] redefined the taxon name Dracomorpha of Northcutt [48] to refer to the crown clade of Iguania + Anguimorpha.

Thus far only a limited number of exonic genes have been studied in Squamata. Reeder et al. [13] and Gemmel et al. [49] conducted phylogenomic analyses of Squamata based on used 46 and 245 orthologs, respectively. Some studies have used somewhat more orthologs than ours but do not cover all the major lepidosaur clades [6, 36]. The present study—based on 966 orthologs from members of every major squamate lineage except Scincoidea (and the enigmatic Dibamidae, whose position remains highly uncertain [20, 21]), viz., Gekkota, Lacertoidea, Serpentes, Iguania, and Anguimorpha—is one of the largest of its kind. It confirms whole-genome support for the clades Toxicofera and Dracomorpha. But it also highlights extensive gene-tree conflict within Toxicofera, with nearly an identical number of genes for the three alternative hypotheses, when analyzed individually. Less conflict was reported by Singhal et al. [21] between the most-supported topology and its next rivals using ultraconserved elements, anchored hybrid enrichment loci and a few dozen single-copy nuclear genes. The conflict we observe amongst gene trees is likely due to incomplete lineage sorting or limited resolution from single loci. As the support for alternative branches is equally strong, geneflow (introgression) can be excluded.

The gene-tree conflict we observe concerning *Shinisaurus crocodilurus* is consistent with previous studies (e.g., [50]), yet clear support for Paleoanguimorpha is present. We could confirm a sister-group relationship between *Lanthanotus* and *Varanus*, as virtually all studies have determined [13, 20, 21, 40, 43–45, 51–53], but we also find that the divergence time estimates between these lineages to be centered in the early Eocene (c. 53 Ma), with a 95% confidence interval < 70 Ma. While it is almost certain that this divergence occurred before the Eocene, as a fossil member of the *Varanus* lineage is known from the Paleocene-Eocene Thermal Maximum in the earliest Eocene [54], the inferred position of various taxa from the Cretaceous of Mongolia is inconsistent with our results. Specifically, *Cherminotus longifrons* has been considered a possible stem representative of *Lanthanotus* [42, 55]. In a recent study, not only did Dong et al. [56] recover this relationship, but they also inferred that a variety of other Mongolian genera (*Ovoo*, *Telmasaurus*, *Aiolosaurus*, *Saniwides*, *Paravaranus*, and *Proplatynotia*) are on the stem of *Varanus*. In contrast, Reeder et al. [13] suggested that the Mongolian genera they included (*Gobiderma*, *Estesia*, *Aiolosaurus*) represent the stem of *Varanus* + *Lanthanotus*. As the Mongolian taxa are all Campanian (c. 83.6–72.1 Ma) [57], our results are inconsistent with the inclusion of the Mongolian taxa on the stem of either *Varanus* or *Lanthanotus*. Notably, only one of these taxa, *Ovoo gurvel* [58], shows the dual lacrimal foramen that marks the bifurcation of the lacrimal duct posterior to the antorbital wall and is distinctive of both *Varanus* and *Lanthanotus* [40, 50, 52].

Our study further confirms the conclusion that all the major lineages of Squamata diverged in rapid succession within the first 10% of squamate evolutionary history [46]. Squamata originated around the Triassic-Jurassic boundary, and all major lineages (Gekkota, Lacertoidea, Serpentes, Iguania, Anguimorpha) appear by the Early Jurassic. The rapid succession of divergences recovered here would probably further be exacerbated if we had been able to include a member of Scincoidea, which likely diverged between Gekkota and Lacertoidea (e.g., [20, 44, 45]). This is undoubtedly a factor that has complicated the interpretation of squamate evolutionary history, because there was little time between successive divergences for the accumulation of evolutionary changes, and much opportunity during the subsequent 180 Ma for changes to be overprinted.

Other divergence time estimates are in line with previous studies. Jones et al. [28] inferred slightly younger dates, with the basal divergence in Squamata at 193 Ma and a somewhat greater spread for major clades, with Anguimorpha diverging from Serpentes (i.e., no monophyletic Dracomorpha) at 150 Ma. Zheng and Wiens [45] inferred the basal divergence in Squamata at 205 Ma and the divergence of Anguimorpha and Iguania at 182 Ma. Simões et al. [59] and Tałanda et al. [60], using a substantially different data set, also inferred that the basal divergence in Squamata occurred in the latest Triassic. Bolet et al. [61], using a post hoc dating method based on a different morphological matrix, inferred slightly older basal divergences in Squamata at 212 Ma, while Brownstein et al. [62] inferred the basal divergence in Squamata to be at 201 Ma, similar to Benson et al. [63]. Overall, our estimate of the basal divergence in Squamata, at 207 Ma, lies within previous results.

### *Lanthanotus* and the Toxicofera hypothesis

*Lanthanotus borneensis* is a central species for evaluating the Toxicofera hypothesis [17, 64, 65], which is a set of three related claims: (1) Iguania, Anguimorpha, and Serpentes are each other’s closest living relatives amongst squamates, (2) the complex venom glands in the upper jaw of many advanced snakes (Caenophidia) and in the lower jaw of certain anguimorphs (*Varanus*, *Lanthanotus*, *Heloderma*) are homologous to the circumoral glands in the upper and lower jaws of many iguanian lizards, and (3) a set of proteins was recruited in the ancestral toxicoferan circumoral glands and continues to be expressed there in extant Toxicofera [17, 65]. As the sister taxon of *Varanus*, *Lanthanotus* and its genome is critical for understanding the evolution of both venom glands and the proteins expressed within them [66, 67].

The present analyses confirm the first statement (see above). At the same time, our analyses reveal that the extent of conflict between gene and species trees in Toxicofera has been inadequately appreciated. Amongst 966 loci, we find nearly equally balanced support for the three alternative topologies, a phenomenon we largely attribute to incomplete lineage sorting due to the rapid diversification, yet the overall support for the consensus tree is strong, considerably higher than in some previous analyses [6]. Accordingly, while a definitive answer concerning the species tree (such as the reality of the Iguania + Anguimorpha clade = Dracomorpha) is important, the study of any particular anatomical or physiological feature should consider the individual gene trees. It is possible for Serpentes, for instance, to share a derived trait with Iguania that it does not share with Anguimorpha, even if Iguania and Anguimorpha are sister taxa, a phenomenon called hemiplasy [68]. For instance, cystatins have been reported in Serpentes and Anguimorpha only [30]. Additionally, whereas serous glands are found in both the upper and lower jaw of most Iguania, they are found only in the upper jaw of Serpentes and in the lower jaw of Anguimorpha [17, 65, 69]. This was interpreted as showing that serous glands evolved once in both the upper and lower jaws of the ancestral toxicoferan, are retained by Iguania, and were subsequently lost in Serpentes in the lower jaw and by Anguimorpha in the upper jaw, but hemiplasy offers an alternative explanation.

The expression patterns and physiological role of proteins sometimes associated with squamate venoms must be carefully assessed in order to avoid bias [70]. Whereas certain C-type lectins are indisputably associated with toxins in the venom gland of advanced snakes [30–32, 70], others have wider expression patterns in body tissues [70]. Our results do not provide evidence for a specific relation of C-type lectins that were positively selected for on the branch subtending Toxicofera to snake venom proteins.

In summary, future work on the evolution of the venom delivery system in Squamata must consider incomplete lineage sorting and its relevance for homology amongst the components of the venom delivery system. Part of this work should involve the ontogeny of the diverse circumoral glands in squamates, including supralabial, infralabial, dental, and venom glands, which remains poorly understood [71]. Additionally, it would be important to establish the molecular patterning mechanisms that underlie the distribution and evolution of these glands in Toxicofera.

Our work furthermore highlights an unexpected aspect of evolution in Toxicofera. Titin—a key structural component of striated muscle associated with elasticity and other properties—was subject to positive selection on all major branches within Toxicofera (Table 2). This suggests that the evolution of skeletal muscle may have played a key role in the origin and diversification of these squamates, and that the structural basis of locomotory adaptations in Iguania, Anguimorpha, and Serpentes requires new scrutiny. In particular, the positive selection experienced on the stem of the clade we identified as “Serpentes” was extreme, with *ω* > 1000 and an EBF of 1564, an order of magnitude higher than for any other orthogroup examined (Additional files 3–7). Critically, it should be noted that our snake sample did not include members of basal snake lineages, only members of the wide-gaped snakes (Constrictores [72] and Caenophidia) capable of swallowing massive prey (e.g., [73, 74]). In fact, the basal one or two lineages of extant snakes comprise small, fossorial forms specialized on invertebrates. Hence, our results do not point unambiguously to positive selection in titin associated with crown Serpentes, but rather associated with wide-gaped snakes. Notably, the ability of such snakes to swallow extremely large prey depends in part on the ability of their intermandibular muscles to stretch far beyond what is typically possible, and to return to the passive state [75]. Adaptation in titin could have played a decisive role. Furthermore, the protein collagen, an important structural component of the integument and other tissues, was also subject to strong positive selection on the same branch. Molecular adaptation in titin and collagen may have been critical in the evolution of wide-gaped snakes. To further test this proposition, a key limitation of the present study (the lack of genomes of more basal snake taxa) must be addressed in future analyses.

### Population history of *Lanthanotus borneensis*

The effective population size of *Lanthanotus borneensis* peaked between 1 and 2 Ma, declined thereafter and has been low but stable for the past 75 kya (Fig. 4). Strictly speaking, the demographic history we calculated reflects only the population from which the individual was sampled, and further individuals are necessary to infer a broader-scale history. Still, Borneo is part of the province of Sundaland, which encompasses Sumatra, Java, and the Malay peninsula as well as the shallow ocean shelf that connects them. Sundaland was repeatedly exposed during the Quaternary, inducing vast changes in the distribution of various habitat types, including lowland evergreen rainforest [76–78]. These changes were felt across Sundaland.

Because *Lanthanotus borneensis* is a lowland rainforest specialist [11], it would be a reasonable hypothesis to link its demographic history, or that of a particular population, to the changing distribution of lowland rainforest during the Quaternary. Yet, there are serious objections to this hypothesis. There were four previous sea-level highstands as extensive as today’s highstand [79] and many other large-scale habitat fluctuations during the past 1 Ma, but none of these seems to have left any mark on the population size of *L. borneensis*. More specifically, whereas the spatial extent of lowland rainforest on Borneo itself is thought to have changed little since at least 120 ka, lowland rainforest was far more extensive across Sundaland during this time [77]. Finally, the proportional change in the extent of lowland rainforest is much lower than the proportional change in the effective population size in *L. borneensis* since its peak.

If Quaternary glacial cycles left no mark on the effective population size of *Lanthanotus borneensis*, then why not? Cannon et al.’s [76] modeling efforts suggest the possibility that lowland rainforest might have been restricted on Borneo during glacial maxima as “transitional hill forest” expanded, so that the true fluctuations in the extent of lowland rainforest may have been more subdued, and the geographic distribution of *L. borneensis* might have simply shifted back and forth over time.

Moreover, minor fluctuations in population size might be dwarfed by the decrease in size from its peak 1–2 Ma. Alternatively, it could be that the distribution of *L. borneensis* is restricted by other factors, such that the expansion of lowland rainforest across Sundaland did not lead to a concomitant increase in population size.

Finally, it is possible that the observed peak in effective population size reflects a time when the lineage was far more widely distributed than it is today, including potentially mainland southeast Asia. Study of fossils from Southeast Asia could help to test that hypothesis.

The genetic diversity of the here compiled *Lanthanotus borneensis* genome, measured in heterozygous sites per base pair (H_O_), revealed a rather low level of diversity (0.0004), comparable only to the H_O_ for *Heloderma charlesbogerti* (0.0001, Anguimorpha [36]) while being an order of magnitude lower than other squamate genomes like *Cryptoblepharus egeriae* (0.007, Scincidae [80]), *Lepidodactylus listeri* (0.005, Gekkota [80]), and *Pantherophis guttatus* (0.003, Serpentes [81]).

This low level of genome-wide heterozygosity corroborates our PSMC results given that heterozygosity usually also reflects the demographic past of a population [82], suggesting that the population indeed remained small but stable over longer periods of time (~ 75 kya according to the PSMC model, certainly encompassing the last glacial maximum and subsequent deglaciation and associated sea-level changes). Such low levels of genome-wide heterozygosity are commonly considered as indicative for a lowered adaptive potential towards changing environments (e.g., [83]), although notable exceptions exist [84] and might not apply here given the known fluctuations in the habitat of *Lanthanotus borneensis*.

## Conclusions

The earless monitor lizard *Lanthanotus borneensis* was first described in 1878 by Franz Steindachner in Vienna [85]. It was included by Camp [39] in his classification and was central to the monograph of McDowell and Bogert [8] on snake origins, although at the time it was known from fewer than ten specimens, all from Sarawak, Malaysia [1], despite a generous standing offer of reward. In 1961, Tom and Barbara Harrisson of the Sarawak State Museum, while exploring Borneo, discovered a new population near the Niah Caves. They published observations of the living animals and distributed living specimens to select natural history museums around the world, including the Senckenberg Museum of Natural History [9]. After the turn of the millennium, a few further contributions were made to the understanding of the species distribution (reviewed in [3]), culminating in the report of a population in Kalimantan, Indonesia [86]. Following these reports and the appearance of *L. borneensis* in the pet trade in the mid-2010s [11], Langner [3] made an important survey to characterize the habitat and natural history of the species.

*Lanthanotus borneensis* is currently included on CITES Appendix II (reviewed in [11]). The species has been documented from around a dozen localities in total, but it could not be rediscovered at many of these in subsequent surveys [11]. Additional sources of information on the natural populations of *L. borneensis* are therefore highly desirable. Our assessment of effective population size using the PSMC method makes a novel contribution to this effort. The low genome-wide heterozygosity and the low but stable effective population size during the large-scale habitat fluctuations on Sundaland in the Quaternary suggest that *L. borneensis* has an unexpected resilience to environmental perturbations but also a potentially lowered adaptive potential.

Our analysis of 966 orthologous genes representing all major squamate lineages except Scincoidea is one of the largest of its kind and confirms the broad structure of squamate phylogeny [12, 16, 17, 44], as supported also by transcriptomes [19], noncoding ultraconserved elements [14, 15], and anchored hybrid enrichment loci [20]. While confirming the monophyly of Toxicofera, our study highlights extensive incomplete lineage sorting amongst the three main lineages comprising that clade (Serpentes, Anguimorpha, and Iguania), probably due to their rapid divergence. It therefore indicates that a consideration of gene trees and hemiplasy will be critical for further evaluating the Toxicofera hypothesis [17, 65].

## Methods

### DNA extraction and sequencing

Genomic DNA was isolated from 25,000 μL of an EDTA blood sample collected by a veterinarian from the caudal vein of a captive, wild-born, male *Lanthanotus borneensis* specimen [87] (Fig. 1a). The sample was obtained from a German non-commercial breeder by a licensed veterinarian and frozen at − 80 °C. DNA extraction was performed using the Qiagen Genomic Tip 20/G (Hilden, Germany), following the manufacturer’s “Preparation of Blood Samples” protocol. The quantity and purity of the extracted DNA were assessed using a NanoDrop 2000 spectrophotometer (Thermo Fisher Scientific, USA) and electrophoretic profiling on a TapeStation 2200 (Agilent Technologies, USA). A PacBio library was prepared according to the “Preparing SMRTbell® Libraries Using Express Template Prep Kit 2.0 with Low DNA Input” protocol and sent to the Radboudumc Genome Technology Centre (RGTC) in Nijmegen, the Netherlands. One SMRTcell sequencing run was performed in CLR mode on the PacBio Sequel System II (Pacific Biosciences, USA) using the Sequel II Sequencing Kit 2.0, targeting a minimum of 80 × genome coverage for high-accuracy long-read assembly. The fragment size distribution and concentration of the PacBio library were assessed using the TapeStation 2200 (Agilent Technologies) and the Qubit fluorometer (Thermo Fisher Scientific, Waltham, MA), respectively. Illumina short reads (we aimed for 70 × coverage) were produced by NovoGene GmbH on a Novaseq 6000 platform.

### Genome assembly and annotation

A de novo assembly was constructed using the PacBio CLR long reads and Illumina paired-end short reads. Initial assembly was conducted with Miniasm v0.3_r179 [88], following read overlap detection using Minimap2 v2.14 [89] with the “map-pb” preset. The resulting assembly was polished with two rounds of Racon v1.3.1 [90], each preceded by realignment of the PacBio reads to the intermediate assembly with Minimap2.

Subsequently, two rounds of polishing were performed with Pilon v1.23 [91], using the Illumina paired-end reads. Reads were aligned to the intermediate assembly using BWA-MEM v0.7.17 [92], and alignments were sorted and indexed using Samtools v1.9 [93]. Pilon v1.23 [91] was run with default parameters using the “–frags” option.

Assembly quality was evaluated at each step using QUAST v5.0.2 [94] and Qualimap v2.2.2a [95]. Completeness of each assembly was assessed using BUSCO v 4.0.6 using the sauropsida_odb10 set from OrthoDB [23]. A contamination screening and subsequent filtering was performed with the BLOBTOOLSKIT v4.4.5 [24, 96] by combining the long-read mapping file, BUSCO results and a genome-wide BLASTN v2.12.0 + [97] search against the nt database (accessed: 15.05.2025) using the “-max_target_seqs 10,” “-max_hsps 1,” “-evalue 1e-25” arguments.

To annotate repetitive elements, the assembly was first soft-masked using RepeatMasker v4.1.5 (http://www.repeatmasker.org) with the built-in *Anopheles* repeat library (–species anopheles, -xsmall). The masked genome was subsequently used to construct a RepeatModeler database with RepeatModeler v1.0.11 (http://www. repeatmasker.org/RepeatModeler/) using default parameters to identify novel repeat families. The resulting consensus library was then used in a second round of RepeatMasker to annotate repeats comprehensively. All RepeatMasker runs included GC content calculation (-gccalc).

Gene annotation was performed using MAKER v2.31.8 [98, 99]. As input, a soft-masked version of the final genome assembly was provided. Protein homology evidence was supplied in the form of RefSeq protein annotations obtained from NCBI for the following reptile genome assemblies: *Anolis carolinensis* (AnoCar2.0, GCF_000090745.2), *Pogona vitticeps* (pvi1.1, GCF_900067755.1), *Python bivittatus* (Python_molurus_bivittatus-5.0.2, GCF_000186305.1), and *Ophiophagus hannah* (OphHan1.0, GCA_000516915.1). Repeat masking employed the built-in simple model (model_org = simple) along with the default transposable element (TE) protein database distributed with MAKER2 (repeat_protein = te_proteins.fasta). Ab initio gene prediction was conducted using Augustus v2.5.5, configured with the *Homo sapiens* gene models. tRNA prediction was disabled (trna = 0), and ab initio prediction was restricted to unmasked regions only (unmask = 0). General MAKER2 settings included an increased maximum DNA chunk size of 100,000 bp (max_dna_len = 100,000) and an expanded expected maximum intron size of 10,000 bp (split_hit = 10,000), optimizing the annotation process for large eukaryotic contigs.

### Phylogenetic analyses

Our study included a total of 23 species with published genomes (Additional file 1: Table S1) in addition to the focal species, *Lanthanotus borneensis*. We used the American alligator (*Alligator mississippiensis*) and the painted turtle (*Chrysemys picta*) as outgroups to the Lepidosauria. Lepidosaur ingroups include the Tuatara (*Sphenodon punctatus*), three members of Gekkota (*Sphaerodactylus townsendi*, *Gekko japonicus*, and *Paroedura picta*), and three members of Lacertoidea including Teiidae (*Salvator merianae*) and Lacertidae (*Podarcis muralis*, *Lacerta viridis*, and *Zootoca vivipara*). Of the toxicoferan clades, Iguana is represented by three species including both Acrodonta (*Pogona vitticeps*) and Pleurodonta/Iguanidae (*Anolis carolinensis* and *Sceloporus undulatus*). Anguimorpha is represented by four species including both Neoanguimorpha (*Ophisaurus gracilis*) and Paleoanguimorpha (*Shinisaurus crocodilurus*, *Varanus komodoensis*, and *Lanthanotus borneensis*). Serpentes is represented by seven species including Constrictores sensu Georgalis and Smith [72] (*Python bivittatus*) and Caenophidia (*Crotalus viridis*, *Protobothrops mucrosquamatus*, *Thamnophis sirtalis*, *Laticauda colubrina*, *Notechis scutatus*, and *Pseudonaja textilis*).

Of the major lepidosaur clades, only Scincoidea was not represented by a genome in our study. Additionally, it is noteworthy that no genome of an early-diverging scolecophidian lineage is available.

Phylogenomic reconstructions were performed using the GeMoMa-to-Phylogeny wrapper function as presented in further detail in Wolf et al. [100]. Publicly available genome assemblies (Additional file 1: Table S1) as well as the genome constructed here were annotated based on information retrieved from the respective public genome repositories (Additional file 1: Table S1) using the GeMoMa v1.7.1 pipeline [101]. Resulting annotations were used in ortholog calling with OrthoFinder v.2.5.2 [102] and we extracted single-copy orthologous sequences (SCOS) with not more than 25% missing species. Orthologous sequences were aligned with Mafft v7.475 applying 1000 iterative refinements. Alignments were trimmed using ClipKit v1.1.3 in “kpic-smart-gap” mode to allow for an additional smart-gap-based trimming. Based on the trimmed alignments, gene trees were constructed with IQtree v2.1.2 [103, 104] with 1000 bootstrap replications each. We further filtered gene trees and alignments based on the maximum likelihood genetic distance calculated by IQtree. To do so, we removed orthologs in the 5% and 95% quantiles to avoid taking misalignments into account as well as sequences with too little information for a meaningful tree construction. Subsequently, all alignments were concatenated using FASconCAT-G v1.04 [105] and an overall tree was compiled with IQtree using the same 1000 bootstrap replications. Additionally, Astral-III v5.7.3 [25] was used to create a consensus tree based on all individual gene trees which also performed quartet score calculation to assess the amount of genetic conflicts within the dataset.

Tree calibration of the concatenated overall tree was performed using IQtree’s implementation of the least square dating (LSD2) method [106, 107]. This method seeks to minimize the squared difference between the observed genetic distances and the expected genetic distances calculated from the divergence times (in Ma) and the substitution rate. The method is considered to be robust to violations of the molecular clock. Confidence intervals are calculated by resampling branch lengths (genetic distances) a specified number of times. In this method, the constraints are treated as fixed and therefore have no confidence intervals. We calculated divergence times given the constraints as described below with 100 replications to estimate confidence intervals.

Minima see [108] are therefore not desirable to use for constraints. Thus, we employed constraints considered close to the true divergence ages, or generous maxima. Furthermore, we are especially concerned with the divergence between *Lanthanotus* and its sister taxon *Varanus*. Therefore, we avoided constraints within the focal clade Anguimorpha. This allows us to compare our results independently with the known anguimorph fossil record. Specifically, in our main analysis, we set minimum divergence times for the following four clades: Diapsida (the crown group of Sauropsida, to which all species included in our phylogenomic analysis belong), Lepidosauria, Lacertini, and Caenophidia as detailed in Table 3. A minimum age on the basal divergence of Diapsida is given by Late Permian *Protorosaurus speneri*, and we take the explicitly exaggerated (soft) maximum age of 295.9 Ma from Benton et al. [27], who emphasized the Middle Permian gap in the fossil record. A minimum age on the basal divergence of Lepidosauria is given by Early Triassic cf. *Diphydontosaurus* [28], and we take the (soft) maximum age of 252.7 Ma, just prior to the Permian–Triassic boundary; this age was detailed by Benton et al. [27] and is close to modern reconstructions. A minimum age on the basal divergence of Colubroidea is given by the late Eocene “Colubrid Indet. MPH” [109, 110]. Although a mid-Eocene age (c. 47 Ma) has sometimes been estimated for Colubroidea e.g., [111], given the probable deeply nested position of Colubrid Indet. MPH [109], we use a more generous maximum at the Paleocene-Eocene boundary. A minimum age on the divergence of *Podarcis muralis* from other lacertids is given by *Lacerta* ex. gr. *L. viridis* from the early Miocene of the Czech Republic [112]. The basal divergence of the earliest branch of the sister group, Gallotiinae, was estimated to be in the early Oligocene [113], and fossils are consistent with that estimate [114], so we use Hipsley et al.’s [113] estimate around the Eocene–Oligocene boundary for a constraint.

**Table 3:** Divergence times estimated for the calibration points used in tree calibration

Clade name (composition)	Fossil calibration for minimum	Age used	Fossil references
Diapsida (Archosauria + Lepidosauria)	*Protorosaurus speneri*	295.9 Ma	Benton et al. 2015
Lepidosauria (*Sphenodon* + Squamata)	cf.* Diphydontosaurus*	252.7 Ma	Jones et al. 2013; Benton et al. 2015
Lacertini (*Lacerta* + *Podarcis*)	*Lacerta* ex. gr.* L. viridis*	33.9 Ma	Čerňanský 2010; Hipsley et al. 2009
Colubroidea (Viperidae + Colubridae)	Colubrid Indet. MPH	56.2 Ma	Smith 2013; Zaher et al. 2019

For a detailed justification of calibrations, see text.

### Effective population size

*Lanthanotus borneensis* begins to breed in the 4th year of life (M. Zollweg, pers. comm.), establishing a minimum generation time that was previously unknown [11]. To establish a maximum generation time, we took length data for the first 4 years of life—total length = (10.0, 15.6, 29.8 cm) at age (in years) = (0, 1, 4)—(M. Zollweg, pers. comm.) and fit a von Bertalanffy equation [115] to the data, solving for the time required to achieve maximum size (*L*), which we took to be 45 cm based on (limited) observations of wild populations [3], rounding up to the nearest centimeter. This yielded an age of 10.6 years (Additional file 2). These values provide reasonable bounds on generation time.

A model about the demographic history of *Lanthanotus borneensis* was compiled using the pairwise sequentially Markovian coalescent (PSMC) framework [116]. This approach aims to reconstruct the effective population size of a diploid species through time by estimating the age at which each pair of heterozygous loci (alleles) diverged (or, looking backwards, “coalesce”) and then leveraging the inverse relationship between the number of coalescent events and population size [116].

Initially, genomic variances were called based on the repeat-masked assembly and the mapped short reads as described above. To do so, BCFtools v1.12 mpileup and call [117] were run with the respective “-c” flag and minimal mapping- and base-quality cutoffs of 25. We further filtered variances for call quality (25) and excess or lack of coverage (< × 3 and > × 0.3 of the mean expected coverage) using BCFtools filter. A consensus sequence was called using BCFtool’s vcfutils.pl. A PSMC model was constructed based on the consensus sequence and a standard of 64 atomic intervals (-p = 4 + 25 × 2 + 4 + 6). Eventually, the model was scaled using the generation time of 10.6 years as described above and a mutation rate of 8.16 × 10^−9^ per site per generation (10.6 years per generation × 7.7 × 10^−10^ per site per year) [118].

Genome-wide heterozygosity was calculated using the same filtered variances that still include monomorphic sites by dividing the number of sites with divergent genotypes by the total number of filtered sites for details see [119]. Due to the low heterozygosity that we calculated, we also re-ran all PSMC analysis using smaller numbers of atomic intervals (34 bins ~ (-p 4 + 10*2 + 4 + 6), and 24 bins ~ (-p 4 + 10*2 + 4 + 6)), which will have larger numbers of coalescent events per bin.

### Positive selection and functional enrichment

To generate nucleotide alignments of the previously compiled set of amino acid orthologous sequences, gff annotations files, created by GeMoMa (see above), and the respective genome assembly fasta files were used to extract nucleotide sequences for all genes per species with gffread v0.12.8 [120]. The sequences corresponding to the final SCOS set (see above) were extracted from these nucleotide fasta files using their gene-IDs and were subsequently aligned using the codon-aware Pal2Nal v.14 [121] which takes their trimmed amino acid counterparts (see above) as reference. Codon-aware nucleotide alignments were trimmed with ClipKit using the "-co" codon mode and subsequently used for positive selection tests using the aBSREL (Adaptive Branch-Site Random Effects Likelihood) model of HyPhy v2.5.62 [122]. Doing so, the rooted maximum likelihood species tree described above was presupposed and different branches were tested for signs of positive selection: Toxicofera, Dracomorpha, Serpentes, Iguania, and Anguimorpha. Since HyPhy demands alignments that contain each species represented in the species tree, the total number of tested genes was reduced to 551 genes. Genes exhibiting significant *p* values for were collected from each branch-test and sorted by likelihood ratio test (LRT) values. To acquire functional annotations, the set of 551 genes were annotated using InterProScan v 5.76–107.0 [123] with the “-goterms” option. A Venn diagram was constructed with ggplot2 v0.1.19. The two genes with the highest LRT, given an empirical Bayes factor (EBF) of more than 5, were reported alongside the tested clades.

A functional enrichment analysis of all genes under positive selection in the Toxicofera clade, given the annotated functions of the 551 single-copy orthologs, was done with the R package topGO v.2.50.0 [124]. All three major ontology categories (molecular function, biological process, cellular component) were tested using the “weight01” algorithm, calculating a “weightFisher” statistic. A Bonferroni (false discovery rate) correction was applied using the p.adjust() function in R. An interactive graph of all three ontology categories was created using the online application REVIGO v1.8.2 [125].

## Supplementary Information

Additional file 1. Supplementary figures and tables. Supplementary figures S1–S4 and supplementary tables S1–S2 with legends.

Additional file 2. Calculation of generation time. R code for calculation of upper limit on generation time based on growth data of captive specimen and maximum size in the wild.

Additional file 3. Positively selected genes on the branch subtending Toxicofera. HyPhy output, indicating positively selected genes by orthogroup identifier, *d*_*N*_/*d*_*S*_ ratio, empirical Bayes factor, likelihood ratio test statisticof model allowing positive selection to null model not allowing it, and annotationand associated gene ontology identifier.

Additional file 4. Positively selected genes on the branch subtending Dracomorpha. HyPhy output, indicating positively selected genes by orthogroup identifier, *d*_*N*_/*d*_*S*_ ratio, empirical Bayes factor, likelihood ratio test statisticof model allowing positive selection to null model not allowing it, and annotationand associated gene ontology identifier.

Additional file 5. Positively selected genes on the branch subtending Iguania. HyPhy output, indicating positively selected genes by orthogroup identifier, *d*_*N*_/*d*_*S*_ ratio, empirical Bayes factor, likelihood ratio test statisticof model allowing positive selection to null model not allowing it, and annotationand associated gene ontology identifier.

Additional file 6. Positively selected genes on the branch subtending Anguimorpha. HyPhy output, indicating positively selected genes by orthogroup identifier, *d*_*N*_/*d*_*S*_ ratio, empirical Bayes factor, likelihood ratio test statisticof model allowing positive selection to null model not allowing it, and annotationand associated gene ontology identifier.

Additional file 7. Positively selected genes on the branch subtending Serpentes. HyPhy output, indicating positively selected genes by orthogroup identifier, *d*_*N*_/*d*_*S*_ ratio, empirical Bayes factor, likelihood ratio test statisticof model allowing positive selection to null model not allowing it, and annotationand associated gene ontology identifiers.

## Data Availability

The datasets supporting the conclusions of this article are available in the Dryad repository, (https:/doi.org/10.5061/dryad.stqjq2cg9) [67]. All sequencing data was uploaded to NCBI and is assigned to the BioProject PRJNA1313344 (https:/www.ncbi.nlm.nih.gov/bioproject/PRJNA1313344), the BioSample SAMN50892088 and raw reads can be accessed via the SRA repositories SRR35210199 (PacBio CLR) and SRR35210198 (Illumina) while the reference genome is assigned the genome ID JBQWDX000000000. All code used to conduct the phylogenetic analyses was published in Wolf et al. [38], and the script to calculate genome-wide heterozygosity was published in Wolf et al. [60].

## Abbreviations

EBF: Empirical Bayes factor
GO: Gene ontology
kya: Thousand years ago
LRT: Likelihood ratio test
Mbp: Million base pairs
Mya: Million years ago
OG: Orthogroup
