# Supplementary material for: The genome of the relict earless monitor lizard, Lanthanotus borneensis, and the Toxicofera hypothesis
Source: BMC Biol. 2026 Mar 5;24:58. doi: 10.1186/s12915-026-02552-4 (PMC12961865; doi:10.1186/s12915-026-02552-4)
Supplement: Supplementary file 1 — Additional file 1. Supplementary figures and tables. Supplementary figures S1–S4 and supplementary tables S1–S2 with legends. [file 12915_2026_2552_MOESM1_ESM.pdf]

# Supplementary file 1

## The genome of the relict Earless Monitor Lizard, *Lanthanotus borneensis*, and the Toxicofera hypothesis

**Magnus Wolf, Axel Janke, Krister T. Smith**

**This PDF file includes:**

Figure S1 to S2

- **Figure S1: Repeat landscape of the Earless Monitor Lizard (*Lanthanotus borneensis*) genome assembly.**
- **Figure S2: Divergence time estimates for Squamata over the last 300 Mya based on nine calibration points and the LSD2 method from IQTree.**
- **Figure S3: Bootstrapped population history of *Lanthanotus borneensis* using 34 bins ~ (-p 4+10*2+4+6).**
- **Figure S4: Bootstrapped population history of *Lanthanotus borneensis* using 24 bins ~ (-p 4+10*2+4+6)**

Table S1 to S2

- **Table S1. Genomic data featured in the phylogenetic analysis of this study.**
- **Table S2. Additional calibration points used for divergence time estimates in Figure S2.**

## Supplementary Figures:

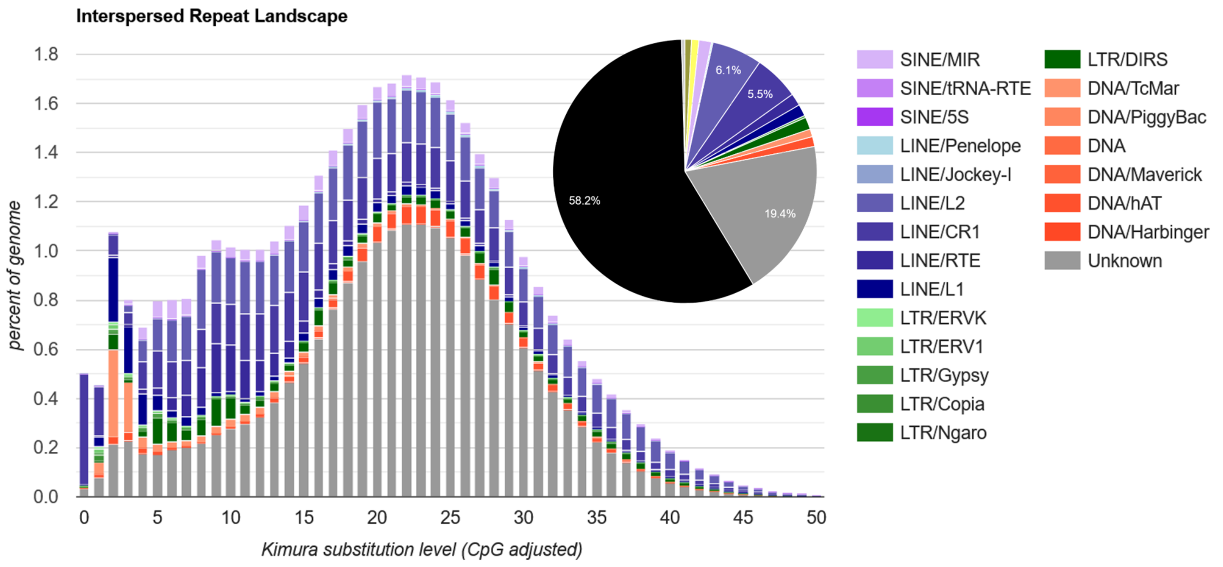


**Figure S1: Repeat landscape of the Earless Monitor Lizard (*Lanthanotus borneensis*) genome assembly.** Colors represent types of repetitive regions, gray areas indicate unclassified types of repetitive regions.

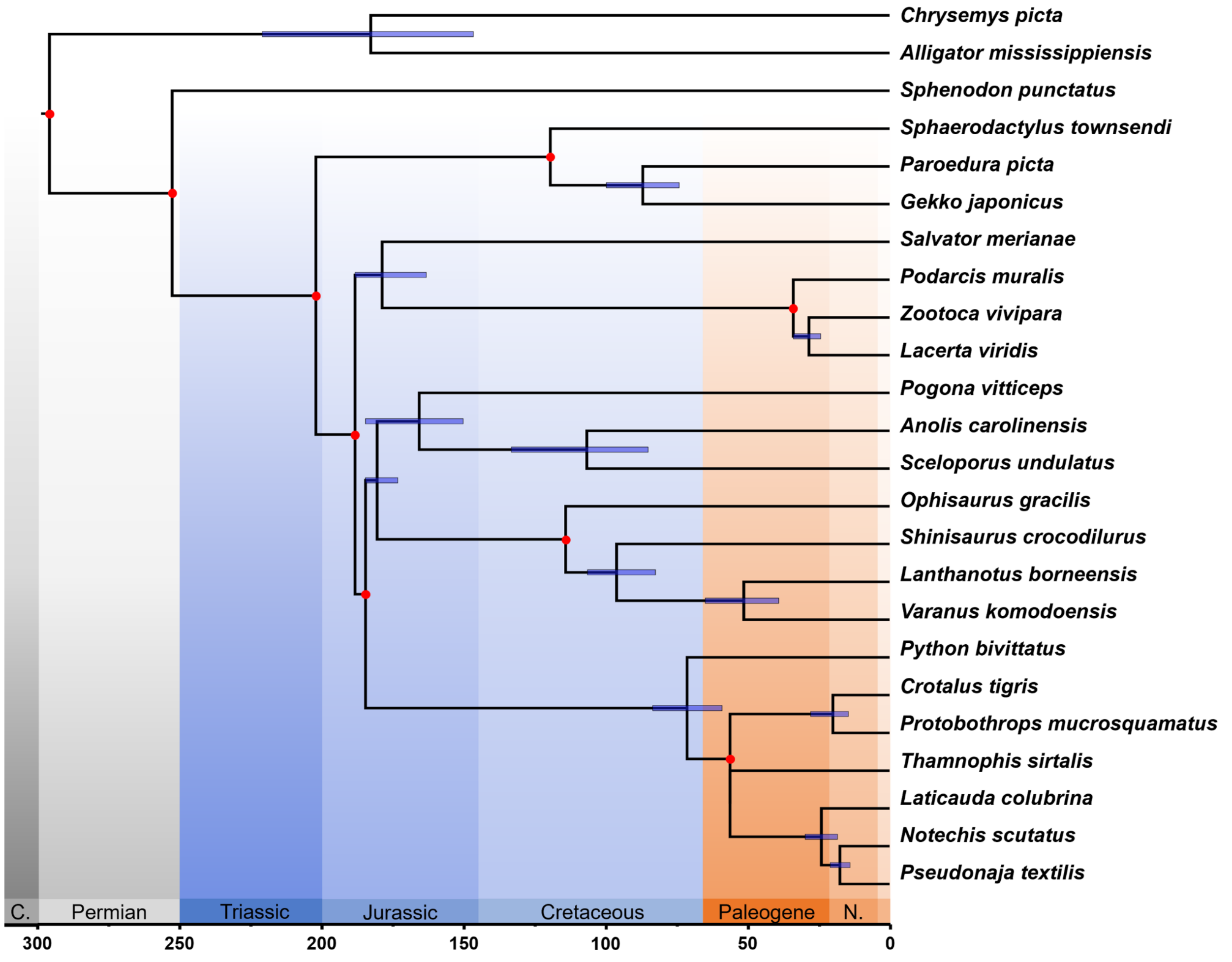


**Figure S2: Divergence time estimates for Squamata over the last 300 Mya based on nine calibration points and the LSD2 method from IQTree.** The used data and methods match Figure 3 of the main manuscript. Calibration data can be found in Table S2.

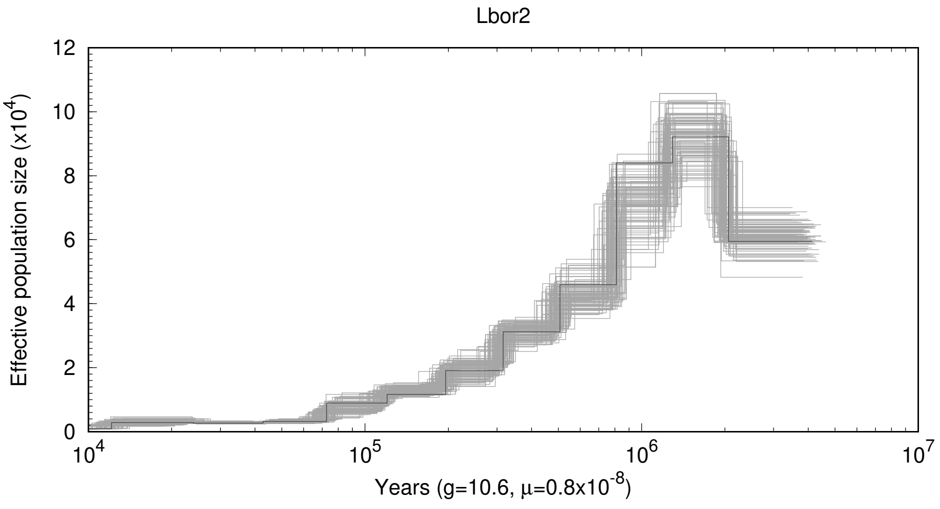


**Figure S3: Bootstrapped population history of *Lanthanotus borneensis* using 34 bins ~ (-p 4+10*2+4+6).** The same pattern is recovered as for the analysis with default bin number (Fig. 4 in main text), although the resolution is lower.

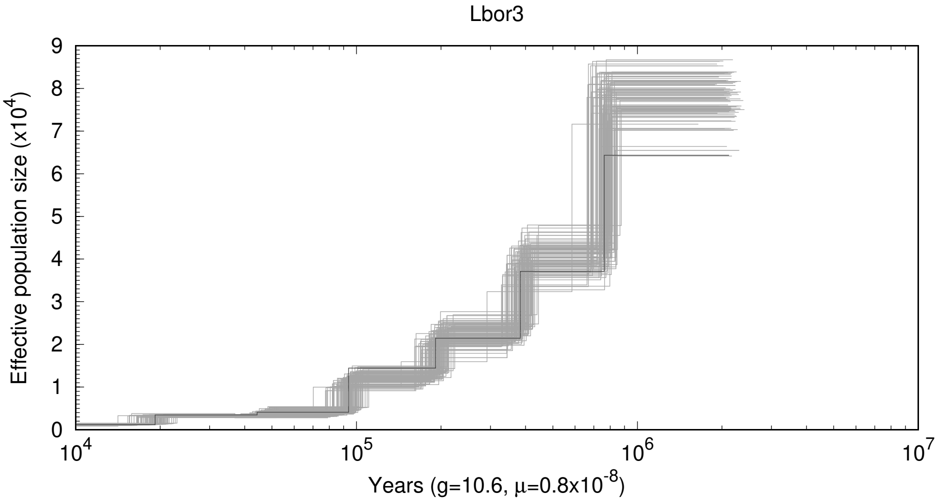


**Figure S4: Bootstrapped population history of *Lanthanotus borneensis* using 24 bins ~ (-p 4+10*2+4+6).** The same pattern is recovered as for the analysis with default bin number (Fig. 4 in main text) and that with 34 bins (Fig. S3 above), although the resolution is lower still.

## Supplementary Tables:

**Table S1. Used genomic data featured in the phylogenetic analysis of this study**. Provided are the scientific name, the Assembly (GeneBank) ID, the respective NCBI BioProject and BioSample ID as well as information on whether the respective annotation was used in the annotation step of GEMOMA-to-Phylogeny.

| Species | Assembly (GenBank-) ID | BioProject | BioSample | Annotation used |
|---|---|---|---|---|
| *Alligator mississippiensis* | GCF_000281125.3 | PRJNA159843 | SAMN02981418 | X |
| *Anolis carolinensis* | GCF_000090745.1 | PRJNA60547 | SAMN02981230 | X |
| *Chrysemys picta* | GCF_000241765.4 | PRJNA210179 | SAMN02713681 | X |
| *Crotalus tigris* | GCF_016545835.1 | PRJNA699292 | SAMN12497667 | X |
| *Gekko japonicus* | GCF_001447785.1 | PRJNA308133 | SAMN04157958 | X |
| *Lacerta viridis* | GCA_900245905.1 | PRJEB24178 | SAMEA104452607 | |
| *Notechis scutatus* | GCF_900518725.1 | PRJNA494227 | SAMEA4800209 | X |
| *Paroedura picta* | GCA_003118565.2 | PRJDB5392 | SAMD00069447 | |
| *Podarcis muralis* | GCF_004329235.1 | PRJNA529705 | SAMN10820182 | X |
| *Pogona vitticeps* | GCF_900067755.1 | PRJNA382134 | SAMEA2300447 | X |
| *Protobothrops mucrosquamatu* | GCF_001527695.2 | PRJNA313429 | SAMD00043515 | X |
| *Pseudonaja textilis* | GCF_900518735.1 | PRJNA495355 | SAMEA4800208 | X |
| *Python bivittatus* | GCF_000186305.1 | PRJNA238085 | SAMN02981298 | X |
| *Salvator merianae* | GCA_003586115.2 | PRJNA473319 | SAMN09273531 | |
| *Sceloporus undulatus* | GCF_019175285.1 | PRJNA746303 | SAMN14373011 | X |
| *Shinisaurus crocodilurus* | GCA_021292165.1 | PRJNA726336 | SAMN19072228 | |
| *Sphaerodactylus townsendi* | GCF_021028975.2 | PRJNA788548 | SAMN20179316 | X |
| *Sphenodon punctatus* | GCA_003113815.1 | PRJNA418887 | SAMN08038466 | |
| *Thamnophis sirtalis* | GCF_001077635.1 | PRJNA294278 | SAMN03759628 | X |
| *Varanus komodoensis* | GCF_004798865.1 | PRJNA523222 | SAMN10967258 | X |
| *Zootoca vivipara* | GCF_011800845.1 | PRJNA638687 | SAMN14329711 | X |

**Table S2. Divergence times estimated for the calibration points used in tree calibration.** The table contains all additional points used in the tree calibration presented in Figure S2.

| Clade name (composition) | Age used | Reference |
|---|---|---|
| Squamata | 202.1 Ma | Zheng and Wiens (2016) |
| Episquamata | 188.3 Ma | Zheng and Wiens (2016) |
| Toxicofera | 184.6 Ma | Zheng and Wiens (2016) |
| *Sphaerodactylus* + *Gekko* | 119.5 Ma | Zheng and Wiens (2016) |
| Anguimorpha | 114.1 Ma | Zheng and Wiens (2016) |
